# Negative Effects of a Nonhost Proteinase Inhibitor of ~19.8 kDa from *Madhuca indica* Seeds on Developmental Physiology of *Helicoverpa armigera* (Hübner)

**DOI:** 10.1155/2014/202398

**Published:** 2014-09-14

**Authors:** Farrukh Jamal, Dushyant Singh, Prabhash K. Pandey

**Affiliations:** Department of Biochemistry, Dr. Ram Manohar Lohia Avadh University, Faizabad 224001, India

## Abstract

An affinity purified trypsin inhibitor from the seed flour extracts of *Madhuca indica* (MiTI) on denaturing polyacrylamide gel electrophoresis showed that MiTI consisted of a single polypeptide chain with molecular mass of ~19.8 kDa. MiTI inhibited the total proteolytic and trypsin-like activities of the midgut proteinases of *Helicoverpa armigera* larvae by 87.51% and 76.12%, respectively, at concentration of 5 *µ*g/mL with an IC_50_ of 1.75 *µ*g/mL against trypsin like midgut proteinases. The enzyme kinetic studies demonstrated that MiTI is a competitive inhibitor with a *K*_*i*_ value of 4.1 × 10^−10^ M for *Helicoverpa* trypsin like midgut proteinases. *In vivo* experiments with different concentrations of MiTI in artificial diet (0.5, 1.0, and 1.5% w/w) showed an effective downfall in the larval body weight and an increase in larval mortality. The concentration of MiTI in the artificial diet to cause 50% mortality (LD_50_) of larvae was 1.5% w/w and that to cause reduction in mass of larvae by 50% (ED_50_) was 1.0% w/w. Nutritional indices observations suggest the toxic and adverse effects of MiTI on the growth and development of *H. armigera* larvae. The results suggest a strong bioinsecticidal potential of affinity purified MiTI which can be exploited in insect pest management of crop plants.

## 1. Introduction

*Helicoverpa armigera* (Lepidoptera: Noctuidae) commonly known as cotton bollworm or American bollworm is a major pest of many important crop plants (>180 plant hosts from >45 families) causing heavy crop losses every year to agricultural, horticultural, and ornamental crops [1]. It is a major polyphagous and cosmopolitan pest, widespread in central and southern Europe, temperate Asia, Africa, Australia, and Oceania, and has also recently been established successfully in Brazil [2]. Larvae of* H. armigera* are foliar feeders as early instars and later shift to the seeds and fruits, causing a drastic reduction in yield estimated to be greater than US$2 billion annually.

*H. armigera* frequently develops rapid resistance to insecticides as compared to some other polyphagous pests [3]. Both the host plant and herbivore struggle hard to overcome the defense of each other, and to survive ensuring a parallel coevolution among them [4, 5]. The digestive enzymes especially the proteolytic enzymes play important roles in insect growth, development, and reproduction processes [6] and therefore these enzymes demand attention as a target for insect pest management [7, 8].

Plants have evolved protease inhibitors (PIs) as one of the natural defensive strategies against insect pests. Although, natural PIs are distributed in all living organisms but they are commonly expressed in plants organs [9]. PIs are small regulatory proteins normally present at 5–15% of total protein concentration and the inhibitory activity of PIs is mostly carried out by different molecular interactions involved in stabilization of reactive site structure [10]. Serine proteinases are the main enzymes present in the midgut of lepidopterans [11] and they are responsible for about 95% of total proteinase activities [12]. Serine proteinase inhibitors have gained importance due to their ubiquitous distribution in the plant kingdom [7]. PIs have been extensively studied for development of resistance against insect pest. PIs also manifest as antinutritional agents, especially in insects where they inhibit midgut proteinases [7, 13, 14].

*Madhuca indica* (Mahua) belongs to the family Sapotaceae; an Indian tropical tree distributed in the central and north Indian plains and forests. The tree, its seeds and flowers have been very useful in Indian economy for a long time. The seeds are used for treatment of enlarged axillary gland, neurotic disorder, aphrodisiac in cough, and bronchitis, relief of pain in the muscle and joints to improve the texture, and curing of bleeding gums and ulcers. The present work describes the purification of a bioinsecticidal trypsin inhibitor from* Madhuca indica* seeds and its effect on developmental physiology of the polyphagous insect* H. armigera* through a series of* in vitro* and* in vivo* experiments.

## 2. Materials and Methods

### 2.1. Seed Material and Insect Rearing

*Madhuca indica* mature and pale-yellow ripe seeds were collected from the trees available locally.* H. armigera* larvae for* in vitro* and* in vivo *studies were from a laboratory colony which was obtained from Narendra-Dev University of Agriculture and Technology, Kumarganj, Faizabad, India. Insects were housed at 28 ± 2°C, 60% relative humidity with a photoperiod of 14 h light and 10 h dark. The composition of the artificial diet was similar to that followed by Singh et al. [13].

### 2.2. Chemicals

N-*α*-Benzoyl-DL-arginine-p-nitroanilide (BApNA), soybean trypsin inhibitor (SBTI), Bovine serum albumin (BSA), acrylamide, bis-acrylamide, and other electrophoretic reagents were procured from Sigma Chemical Co. (St. Louis, Mo, USA). Sephadex G-75 was purchased from Amersham Biosciences (Uppsala, Sweden). Trypsin-Sepharose CL-4B, low molecular weight markers and azocasein were purchased from Sisco Research Limited (Mumbai, India). All other chemicals and reagents used were of analytical grade.

### 2.3. Isolation and Purification of Trypsin Inhibitor from* Madhuca indica* Seeds

*Madhuca indica* trypsin inhibitor (MiTI) was purified as described earlier by our working group [13]. Dried seeds were ground to fine powder, depigmented, and defatted with several washes of chilled acetone and hexane. The clear supernatant was collected at 12,000 rpm for 20 min at 4°C and three cuts of (NH_4_)_2_SO_4_ precipitations were done, 0–30% (*F*_1_), 30–65% (*F*_2_), and 65–95 (*F*_3_). The protein pellets of different fractions were dialyzed against 0.1 M phosphate buffer (pH 7.6) using a membrane with a cut off range 12 kDa (Sigma grade) and lyophilized. Of the three pooled fractions, *F*_2_ (30–65%) showing the maximum trypsin inhibition activity was preferred for gel-filtration chromatography on Sephadex G-75 column (100 × 2 cm) equilibrated with equilibration buffer (50 mM Tris-HCl buffer, pH 7.6). The fractions exhibiting highest trypsin inhibition activity were pooled, dialyzed, and lyophilized. The active fractions eluted from gel-filtration column were applied onto a Trypsin-Sepharose CL-4B column (25 × 1.5 cm) preequilibrated with 0.1 M Tris-HCl buffer (pH 7.6), 5 mM CaCl_2_, and 0.1 M NaCl. The bound proteins were retrieved at an initial flow rate of 30 mL/h using 100 mM HCl solution. The fractions of antitryptic peak were pooled and lyophilized for further analysis. Proteinase inhibitory activity [15] and protein content [16] in the aliquot were determined.

### 2.4. Polyacrylamide Gel Electrophoresis

Sodium dodecyl-sulfate polyacrylamide gel electrophoresis (SDS-PAGE) was carried out at 12% as described by Laemmli [17] at room temperature in the absence and presence of *β*-mercaptoethanol (0.1 M). In the wells, 30 *μ*L (50 *μ*g) of affinity purified sample and 20 *μ*L (as such) of marker (using low molecular weight standard protein markers) was loaded. Molecular weight of unknown protein was calculated from the Genei gel-doc fire-reader software.

### 2.5. Extraction of Midgut Proteinases from Larvae of* H. armigera*

To assess the potency of MiTI on proteinases of* H. armigera*,actively feeding fourth instar larvae of* H. armigera* were cold immobilized and killed by decapitation to collect the midguts along with its content [13]. Replicated sets of five guts were maintained and each gut was transferred into chilled polycarbonate tube and homogenized in 0.1 M glycine-NaOH buffer (pH 10.0) followed by centrifugation at 12,000 rpm for 15 min at 4°C. It was ensured that the inhibitory assay was performed in a buffer capable of neutralizing the acid. Supernatant used as the crude enzymes extract and was stored at −20°C until utilized in inhibition assays.

### 2.6. *In Vitro* Proteinase and Proteinase Inhibitory Assay

Total gut proteinase activity was measured by azo-caseinolytic assay [18]. Trypsin-like activities were estimated using the chromogenic substrate BApNA [15]. BApNA is specific for the determination of trypsin activity [19]. For the inhibitory assays, different concentrations of affinity purified inhibitor and soybean trypsin inhibitor (SBTI) were added to the HGP extract (40 *μ*L) and incubated at room temperature (27°C) for 15 min. The residual proteinase activity was determined spectrophotometrically at 410 nm. Inhibitor activity was calculated by the amount of purified sample required to inhibit 50% of trypsin activity, which is considered as one unit of trypsin inhibition and expressed as trypsin inhibitor units per mg seed protein. Results were expressed as % inhibition relative to controls without inhibitor. Increasing concentrations of MiTI were incubated with 40 *μ*L HGP, and enzyme assay was performed. Percentage of inhibition for each inhibitor concentration was used to create a titration curve. IC_50_ value was determined by the help of titration curve. All* in vitro* assays were carried out in triplicates.

### 2.7. Effect of pH and Temperature on Stability of MiTI against HGP

Thermostability of purified inhibitor was determined at different temperatures (10–100°C). In this assay 5 *μ*g/mL of MiTI was mixed with 0.1 M Tris-HCl buffer (pH 7.6) and incubated at various temperatures for 30 min. The stability of inhibitor was also determined at different pHs (2–12) [13]. After incubation at various temperatures and pH, samples were centrifuged and remaining proteinase inhibitor activity was measured against trypsin like midgut proteinases. BApNA was used as a substrate for inhibition activities.

### 2.8. Determination of Kinetic Parameters

In order to determine the inhibition constant (*K*_*i*_), data was plotted according to Dixon [20]. The inhibition constant was determined for trypsin like midgut proteinases, by preincubating the enzyme with concentrations of MiTI (3, 5, 7, and 10 nM). Two BApNA concentrations (0.005 and 0.01 mM) were used. The velocity of enzymatic reaction was expressed as 1/*v* (OD_410_ mM/min/mL)^−1^ and *K*_*i*_ value was determined by intersection of the two lines for each substrate concentration. To determine the inhibition mechanism of MiTI against trypsin like midgut proteinases, the inhibition kinetic data were analyzed by Lineweaver-Burk double-reciprocal plots.

### 2.9. *In Vivo* Activity of MiTI against* H. armigera*

For* in vivo* studies, MiTI was incorporated into the artificial diet at different concentrations (0.5, 1.0 and 1.5% w/w) as suggested by Giri and Kachole [21], while diet without MiTI was used as control. Larval weight was taken on an interval of two days until pupation. 45 larvae were used for each treatment. All the experiments were performed at 28 ± 2°C and 60% relative humidity and the food was changed at every alternate day to minimize the microbial contamination. The effect of MiTI was evaluated at the larval stage on different parameters like weight, growth, mortality and duration of the larval stages. At the pupal stage, we recorded the pupal development duration and malformed pupae; while for the adult stage, the percentage of deformations and fecundity was recorded in every parallel time interval.

Relative growth rate was calculated by using formulae of Farrar et al. [22]. Relative growth rate = [change in body weight/(initial dry weight of larvae × feeding period duration)]. The relative growth rate of larvae in each treatment was calculated for every two days interval. Mean fecundity was determined by the method reported by Singh et al. [13]. Fecundity (%) = [egg numbers of MiTI treated/egg numbers of control] × 100.

### 2.10. Calculation of LD_50_ and ED_50_ Values of MiTI against* H. Armigera* Larvae

Different dosages of MiTI were used to determine LD_50_ (effective dose to kill 50%* H. armigera * larvae) and ED_50_ (effective dose to cause a 50% reduction in larval weight) dosages.

Each treatment had 45 larvae per replication, and there were three replications in a completely randomized design. Observations were recorded on larval mortality and weight of the surviving larvae after three days of maintaining on the artificial diet treated with various dosages of the MiTI. One set of larvae maintained on untreated artificial diet represented control. The mortality and weights of* H. armigera* larvae maintained on control diet were considered as the basis on which the LD_50_ and ED_50_ values were calculated for the larvae maintained with MiTI incorporated diets. The LD_50_ and ED_50_ values were calculated using the log dose-probit analysis [23].

## 3. Nutritional Parameters

Nutritional indices were measured on dry weight basis. After measuring the weight of the fourth and fifth instar larvae, they were exposed to either the MiTI-supplemented diet or the control diet, and the weights of the larvae were recorded at every alternate day until they reached the prepupal stage. The initial fresh diet as well as the diet and feces remaining at the end of each experiment were weighted. The quantity of food ingested was determined by subtracting the diet remaining at the end of each experiment from the total weight of diet provided.

The following formulae were used as reported elsewhere [22] to calculate CI (consumption index), AD (approximate digestibility), ECI (efficiency of conversion of ingested food), and ECD (efficiency of conversion of digested food): ECI = (Δ*B*/*I*) × 100; ECD= [Δ*B*/(*I* − *F*)] × 100; CI = (*I*/*A*) × 100 and AD [(*I* − *F*)/*I*] × 100, where *I* = weight of food consumed, *A* = mean weight of insect over unit time, Δ*B* = change in body weight and *F* = weight of feces produced during the feeding period. The metabolic cost (MC) was calculated as 100-ECD.

## 4. Statistical Treatment of Data

Statistical analyses were carried out using Graph Pad Prism 4 (Graph Pad software version 4.0, San Diego, CA, USA). To detect significant differences in mean larval weights, the pooled treatment data was subjected to two way repeated measures ANOVA (SAS 1999). Comparisons were made for all parameters within the amounts of inhibitor used as well as the different days of larval development. Simple comparisons, where ever appropriate was made using unpaired *t*-tests. Results with *P* < 0.05 were considered to be statistically significant. For all bioassays, means and standard errors (SE) of the insect were calculated for each treatment.

## 5. Results

### 5.1. Evaluation of MiTI Purity (with Each Purity Level)

**Figure 1:**
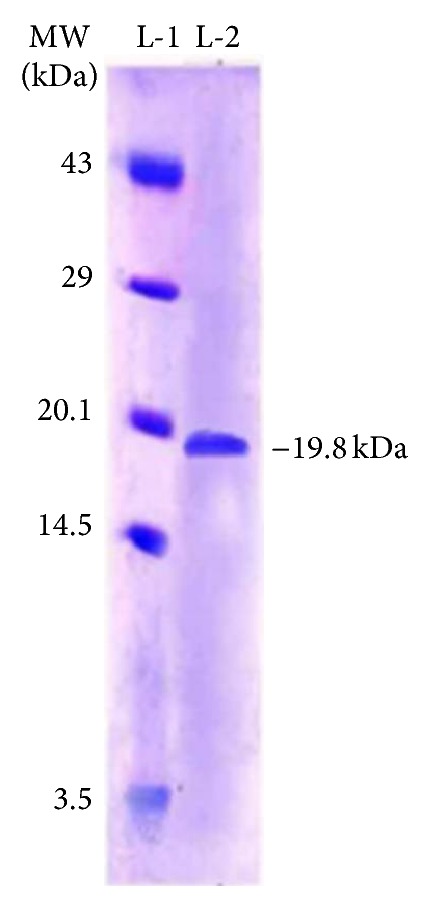
SDS-PAGE showing resolution of fractions: Lane 1 shows the molecular weight marker and Lane 2 shows the separation of affinity purified fraction.

The specific activity, yield performance, and inhibitory activity with each purity level of MiTI have been detailed in Table 1 (Supplementary data available online at http://dx.doi.org/10.1155/2014/202398). During each step of purification the specific activity and percent inhibition of MiTI increased with the decrease in its total yield. The protein eluted on gel filtration represented inhibition (~64%) of midgut trypsin-like activities was loaded on trypsin affinity column (Figure 1(a), Supplementary data). The protein eluted from affinity column represented a considerable inhibition (~76%) against trypsin like midgut proteinases, was pooled and lyophilized (Figure 1(b), Supplementary data). A purification of 3.66-fold with a 15.73% yield was achieved (Table 1, Supplementary data). SDS-PAGE in the presence or absence of *β*-mercaptoethanol showed that MiTI consisted of a single polypeptide chain with a molecular mass of about 19.8 kDa (Figure 1).

### 5.2. Inhibition Assay of HGP by MiTI

**Figure 2:**
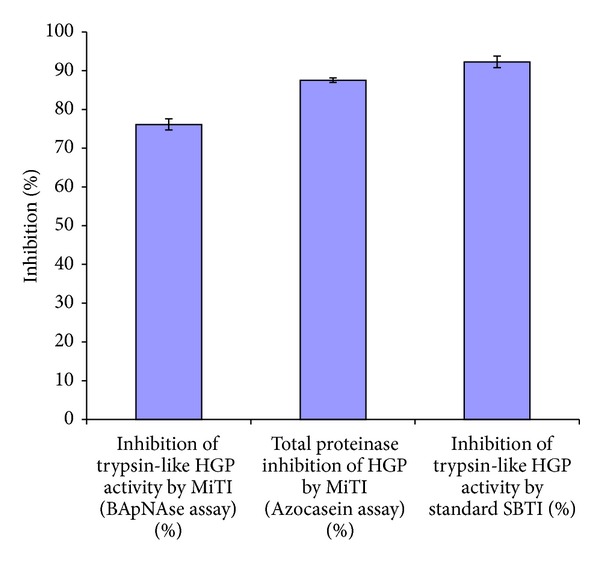
Inhibition of trypsin like and total proteolytic activity of HGP at varying concentrations of inhibitor. The assays were conducted using BApNA and azocasein as substrates. Standard SBTI used as reference standard. Values are mean ± standard error for at least three replications (*P* < 0.05).

**Figure 3:**
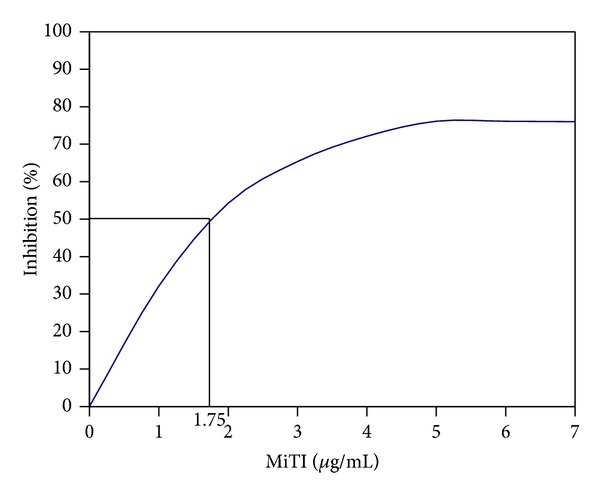
MiTI inhibition curve against trypsin like HGP and determination of IC_50_ value of MiTI. IC_50_ = MiTI concentration which inhibits 50% of trypsin like HGP activity.

Inhibition assays of HGP extract using purified proteinase inhibitor from* Madhuca indica* (MiTI) demonstrated inhibition of HGP. MiTI showed high inhibitory activity towards both trypsin like HGP and total gut proteolytic activity (Figure 2). MiTI (5 *μ*g/mL) inhibited approximately 76% trypsin like and 87.51% total gut proteolytic activity of* H. armigera*. Percent inhibition of trypsin-like HGP activity by standard SBTI was ~92% greater than MiTI activity (Figure 2). The calculated IC_50_ value of MiTI for midgut trypsin like proteinases was 1.75 *μ*g/mL (Figure 3).

### 5.3. *In Vitro* Stability of MiTI at Various pHs and Temperatures

**Figure 4:**
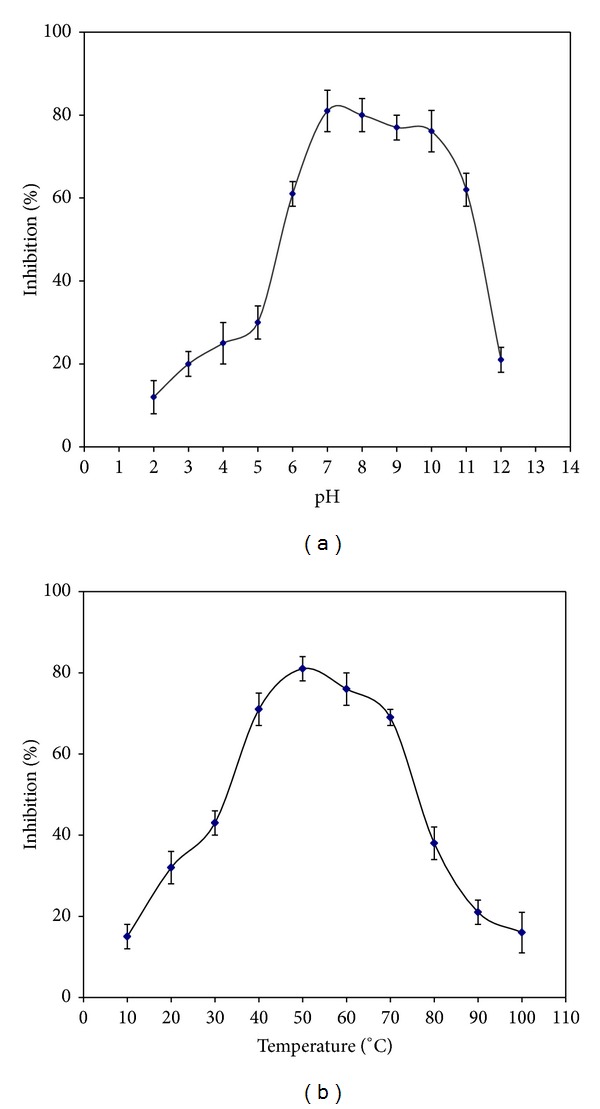
(a) pH and (b) thermal stability profile of purified MiTI. Each point represents the average of three experimental observations. The vertical bar represents standard deviations.

Stability of the inhibitor at different pH (2–12) was determined. MiTI exhibited stability at a pH of 6.0 to 11.0, with maximum stability in a pH range 7 to 8 (Figure 4(a)). Temperature stability at an extensive range of temperatures (10–100°C) was also measured. Results showed that inhibitor was stable up to 60°C. The effectiveness of inhibitor decreased significantly following rise of temperature above 70°C (Figure 4(b)).

### 5.4. Kinetic Determination

**Figure 5:**
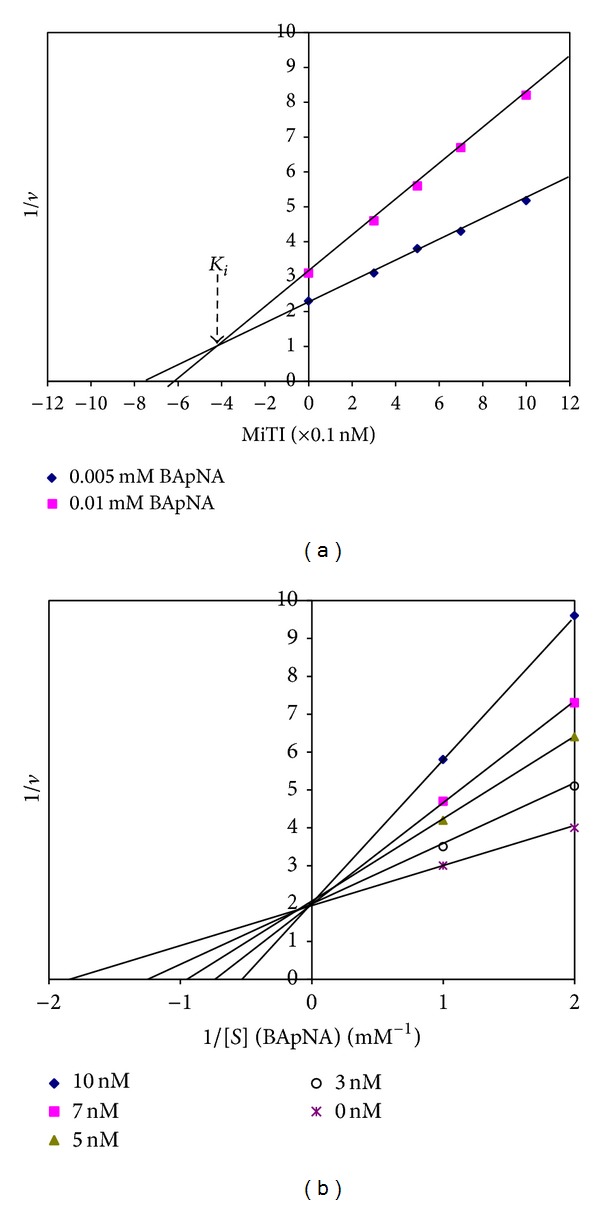
Kinetics analysis of MiTI against trypsin like HGP activities. (a) *K*_*i*_ value was determined by Dixon plot and (b) Lineweaver-Burk plot for determining the nature of inhibition.

Various concentrations of MiTI were assayed in order to determine the mechanism and inhibition constant towards trypsin like midgut proteinases by using various BApNA concentrations as substrate according to Singh et al. [13]. The inhibition kinetic data was analyzed by Dixon and Lineweaver-Burk plots. The kinetic analysis showed that MiTI inhibition of trypsin like midgut proteinases was competitive (Figure 5(b)) with a *K*_*i*_ value of 4.1 × 10^−10 ^M (Figure 5(a)).

### 5.5. Antimetabolic Effect of MiTI on Developmental Physiology of* H. armigera*

**Figure 6:**
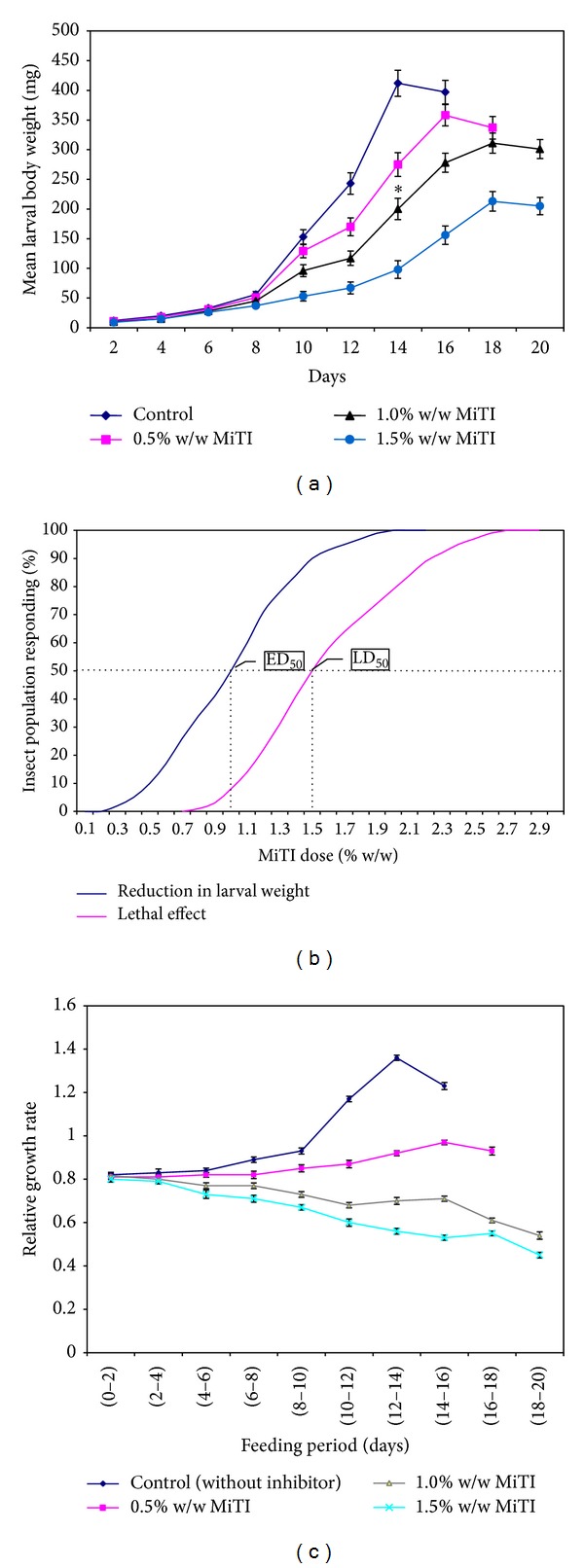
Effect of MiTI on* H. armigera* larval (a) weight (mg), (b) determination of lethal dose and effective concentration for 50% weight reduction by using various concentration of MiTI in artificial diet, and (c) mean relative growth rate. Bars indicate standard error of the mean value. Control was without inhibitor. Calculated LD_50_ and ED_50_ values have been shown.

To estimate the* in vivo* effects of MiTI on growth and development of* H. armigera *larvae, feeding trials were conducted with appropriate controls. Three different doses of inhibitor were incorporated into an artificial diet at levels of 0.5, 1.0, and 1.5 (% w/w). These three concentrations were chosen because the effects of MiTI at these concentrations on larval survival were considered appropriate for* in vivo* experiments. Food intake was drastically reduced in the larvae showing growth retardation. During 0–6 larval development days there was no significant change among various treatments, whereas the reduction in larval weight varied remarkably among different treatment groups during their remaining growth period (Figure 6(a)). Maximum growth retardation was observed in 12–14 larval development days as compared to control. From the above results, it is suggested that the inhibitor concentration of MiTI used in the diet was sufficient to inhibit the growth of larvae.

The LD_50_ of MiTI to* H. armigera* was estimated to be 1.5% w/w and the ED_50_ value of MiTI was 1.0% w/w diet (Figure 6(b)). The estimated LD_50_ and ED_50_ concentrations of MiTI were used to assess the effects on survival, development, and fecundity of the* H. armigera* larvae.

The potential insecticidal effects of MiTI towards the relative growth rate of* H. armigera* larvae were assessed by incorporating different concentrations of the inhibitor in the larval base diet. Relative growth rate was significantly more in the larvae reared on control diet compared to those reared on the test diet. Relative growth rate of* H. armigera* (12–14 development day) larvae on MiTI containing diet has been reported to be significantly lower than in the larvae of other development days. The slowest relative growth rate (0.56) was observed for larvae maintained with 1.5% w/w MiTI concentration against the control (Figure 6(c)).

**Figure 7:**
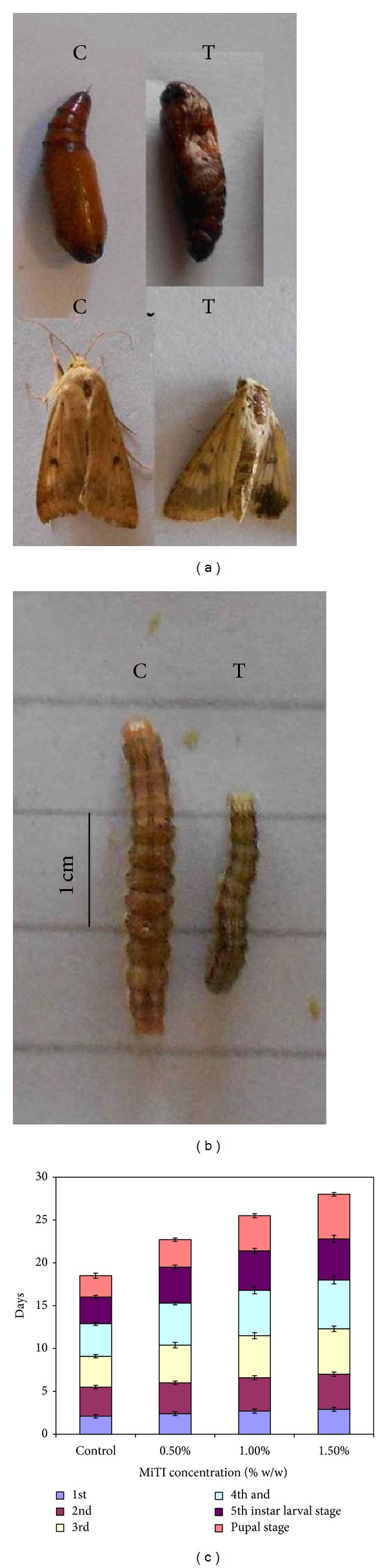
Insect bioassay. Effect of purified MiTI on (a) pupal and adult deformities and (b) larval growth. (c) Effect of MiTI on duration of larval stage and pupal development duration. Values are mean ± standard error for at least three replications. 45 larvae were used for each treatment.

There was a marked reduction in body weight of larvae maintained on MiTI containing diet, whereas larvae on base diet showed better development (Figure 7(a)). Furthermore, duration of the larval development, pupation, and pupal development duration was also delayed and marked by stunted growth (Figure 7(c)). The mean duration of the larval stage on control diet was 16 days and diet containing MiTI (1.5% w/w) was 22.8 days. The mean duration of the pupal stage were 2.5 days in the control diet and 5.2 days in the diet included with MiTI (1.5% w/w).

The pupation rate, adult emergence, deformities and fecundity were also recorded. The MiTI (1.5% w/w) treatment significantly reduced pupation rate and emergence of* H. armigera* larvae. Pupation rate was lowest (67.2%) in larvae reared on diet supplemented with 1.5% w/w MiTI; whereas adult emergence from 1.5% w/w MiTI treated larvae decreased (38.7%) as compared to control larvae. The MiTI containing diets enhanced percent deformities and slowed down the fecundity of* H. armigera*. Among the treated, high percent deformities in pupae and adult insects were observed in larvae reared on diet impregnated with 0.5% w/w (25.8%), 1.0% w/w (37.1%) and 1.5% w/w (52.3%) as compared to control (5.3%) (Table 2 Supplementary data). It is interesting to observe that the mean fecundity was drastically affected in moths emerging from diet containing 0.5, 1.0, and 1.5% w/w MiTI diet (Table 2 Supplementary data). The downfall in mean fecundity was dose dependent. The reduction observed was 62.5% and 49.4% at MiTI concentrations of 1.0 and 1.5% w/w, respectively.

### 5.6. Nutritional Indices

Nutritional indices of fourth and fifth instar larvae of* H. armigera *are provided in Table 3 (Supplementary data). The MiTI fed fourth instar larvae showed higher values of CI (67.87), MC (84.32%), and AD (61.26%), and lower values of ECD (15.68%), and ECI (9.6%) as compared to control diet. CI (58.36%), AD (42.52%), ECI (7.79%), and MC (81.51%) of fifth instar larvae reared on inhibitor containing diet showed lower values as compared to fourth instar larvae. ECD of fourth instar larvae was lower than the fifth instars larvae.

## 6. Discussion

The purification of MiTI was carried out using ammonium sulfate precipitation, subsequent dialysis, gel filtration, and affinity chromatography. In each step specific activity increased with the fold purification. During each step of purification the recovered protein exhibited an increase in inhibitory activity. Purification and inhibitory potential of nonhost proteinase inhibitors have also been reported from various other plants [24–26].

After subsequent assays with MiTI the presence of trypsin inhibitor was confirmed and measured in each purity level. BApNA and azocasein substrates were used to assess the presence and absence of inhibitors in* Madhuca indica *seed extract. Fourth instar larvae were used for* in vitro* experiments because this larval stage exhibits maximum proteinase activity and maximum numbers of proteinase isoforms [27, 28]. As the digestive gut proteinases of lepidopteran insects are optimally active in alkaline medium therefore inhibition assays using HGP extract with MiTI were studied in the basic pH range [29].

Different concentrations of inhibitor extract were used to evaluate the potential of MiTI for inhibiting HGP activity. Standard SBTI used as positive control was highly effective as compared to MiTI against gut enzymes at the equal inhibitor concentration. These findings were also in concurrence with the findings of some other research groups [19, 30]. The results indicated that total inhibitory activity of HGP was more than trypsin like inhibitory activity; suggesting the presence of different proteinases in the gut of* H. armigera*. This higher percent inhibition towards total HGP is possibly an outcome of inhibition of proteinases, other than those which possess trypsin-like activity [31]. Thus, this higher inhibitory activity towards general proteolysis with a nonhost PI would be more attractive in plant defense due to more diverse and detrimental effects on larval growth and physiology [32]. Larval diet containing MiTI showed decreased proteolytic activities of the midgut. The toxic effect of the inhibitor slows down larval midgut proteinase activity by blocking the enzymes engaged in the process of digestion. Inhibitors extracted from chickpea (P-256) inhibited 66%* H. armigera *midgut trypsin activity [33]. Inhibition of more than 80% of* H. armigera* proteinase activity has also been reported with nonhost PIs from bitter gourd [34]. Similarly inhibitors isolated from the seeds of* Prosophis juliflora* exhibited 83% inhibition against* H. armigera* midgut trypsin-like activity [35]. The IC_50_ of MiTI for trypsin like midgut proteinases was 1.75 *μ*g/mL, which was less effective than standard SBTI (0.13 *μ*g/mL) [31, 32].

Affinity purified MiTI was stable in a broad range of temperature and pH. Inhibitory activity of MiTI exposed to different pH, was stable predominantly in alkaline conditions (pH 6.0–11). The optimal pH for digestive proteases in most lepidopteran larvae falls in alkaline condition with a maximal pH of 10 and 11 [36]. Trypsin inhibitor isolated from pigeon pea and jack fruit seeds were also stable over a wider range of pH from 7–10 and 3–12 respectively [37].

MiTI was also stable over a wide range of temperature (20–60°C) but at higher temperatures the inhibitory activity decreased remarkably. Trypsin inhibitors isolated from oat was stable in a wide range of temperatures from 0 to 100°C [38]. A high stability of PIs is an outcome of hydrophobic interactions of short stretches of hydrogen bonded sheets [39]/disulfide linkages minimizing their conformational entropy and consequently enhances their stability [40].

Serine proteinase inhibitors are capable of high affinity inhibition by competitive or noncompetitive mechanisms [39, 41]. The inhibition constant of MiTI for trypsin like midgut proteinases was 4.1 × 10^−10^ M, suggesting a high affinity of the MiTI for trypsin like midgut proteinases. Similar *K*_*i*_ values were reported for other trypsin inhibitors from* Caesalpinia bonduc* seeds (2.75 × 10^−10^ M) [42],* Derris trifoliate* (1.7 × 10^−10^ M) [39],* Dimorphandra mollis* (5.3 × 10^−10^ M) [43], and* Putranjiva roxburghii* (1.4 × 10^−11^ M) [44]. Kinetic studies have also shown competitive inhibition of trypsin by MiTI.* H. armigera* larvae maintained on MiTI dosage inhibited the larval growth and development. Feeding studies demonstrated that MiTI inhibited the growth of* H. armigera *larvae. Starvation and added stress on gut proteinase expression system to synthesize new and higher amounts of proteinases could be the possible reasons for arrested growth and mortality of* H. armigera* larvae [13, 44]. The inhibitors from chickpea, mungbean, and jambul also showed significant reduction in larval growth of* H. armigera* [13, 45]. MiTI delays larval and pupal developmental periods and causes several deformities in neonates. The duration of pupation increased due to interference of MiTI in the moulting process; consequently the larvae were not able to go into further developmental stages of their life cycle. In general, prolonged pupation times directly affected the survival of pupae.

Deformities of neonatal adults reared on different MiTI containing artificial diets were examined. Low survival rate and reduction in fecundity (number of eggs laid down per female) were observed in MiTI treated larvae. The MiTI was strongly effective in reduction of fertility and fecundity which persisted for successive generations. The fecundity of lepidopteran adults is the most commonly used parameter for determining the effect of larval diet on the adult stage.* In vivo* study suggested that quality of diet influences the effectiveness of the inhibitor [46]. Fecundity of* H. armigera* was severely affected by 0.33% concentration of winged bean PI diet [47]. Telang et al. [34] reported a similar effect on* H. armigera* and* S. litura* by using PI from nonhost source such as bitter gourd in the diet. The tomato PIs also cause dose dependent adverse effects on various developmental parameters of* H. armigera*, most notably on fecundity [28].

Nutritional indices study revealed the antinutritional property of MiTI. ECI is generally a measure of an insect's capability to exploit the food ingested for growth [48]. Along with nutritional indices, ECI may vary with the digestibility of food and the proportional amount of the digestible portion of food, which is converted to body mass and metabolized for energy essential for vital activities [48]. In the present study change in ECD also indicates the increased/decreased proportion of digested food metabolized for energy [49]. A nearby or no change in ECI and ECD values as compared to control point out that ingested biochemicals such as PIs do not exhibit any chronic toxicity [50]. However the nutritional indices of the fourth and fifth instar larvae of* H. armigera* were significantly different as compared to control. Therefore the data obtained for the fourth and fifth instar larvae are not dependable on each other. This fact describes that the nutritional requirements of an insect changes during development and such changes are dependent on food consumption and feeding behavior [51]. The higher ECI value of* H. armigera* for fourth instar larvae as compared to control suggests that they were more capable to convert the ingested food into biomass. As compared to fifth instar larvae, fourth instar larvae had lower value of ECD; implying that they were incapable to turn the digested food into biomass. An increased AD value observed for MiTI-fed larvae was most likely a result of the lower fecal output by treated larvae relative to the control group. This fact was also observed by Mordue and Blackwell [52] and Rayapuram and Baldwin [53].

These results from both* in vivo* and* in vitro* studies clearly showed that the MiTI isolated from the seeds of* Madhuca indica* is a strong and effective proteinase inhibitor against the growth and development of* H. armigera*. Further, this inhibitor protein could be exploited in insect-pest management of food crops.

## Supplementary Material

The data available here is about the inhibitor purification from Madhuca indica (MiTI) based on trypsin-like activity of H. armigera midgut proteinases. Sephadex G-75 gel filtration chromatography follows trypsin-sepharose affinity column. The nutritional indices of Helicoverpa armigera larvae reared on MiTI supplemented diet is also tabulated.
