# Supplementary material for: Negative Effects of a Nonhost Proteinase Inhibitor of ~19.8 kDa from Madhuca indica Seeds on Developmental Physiology of Helicoverpa armigera (Hübner)
Source: Biomed Res Int. 2014 Sep 14;2014:202398. doi: 10.1155/2014/202398 (PMC4179944; doi:10.1155/2014/202398)
Supplement: Supplementary file 1 — The data available here is about the inhibitor purification from Madhuca indica (MiTI) based on trypsin-like activity of H. armigera midgut proteinases. Sephadex G-75 gel filtration chromatography follows trypsin-sepharose affinity column. The nutritional indices of Helicoverpa armigera larvae reared on MiTI supplemented diet is also tabulated. [file 202398.f1.pdf]

Supplementary Data:

Table 1 Summary of inhibitor purification from *Madhuca indica* (MiTI) based on trypsin-like activity of *H. armigera* midgut proteases

Purification steps	Total protein (mg)	Total trypsin inhibitory unit (TIU)	Specific activity(TIU/mg protein)	Yield recovery (%)	Fold purification	Percent inhibition
Crude extract	312.37	553	1.77	100	1.00	39.80
$F_{30-65\%}$ $(NH_4)_2SO_4$ saturation fraction	91.52	264	2.88	47.73	1.62	48.40
Dialysis	42.70	162	3.79	29.29	2.14	56.70
Gel filtration (Sephadex G-75) chromatography	21.30	117	5.49	21.15	3.10	64.60
Trypsin affinity chromatography	13.41	87	6.48	15.73	3.66	76.10

TIU= One inhibition unit is defined as the amount of the inhibitor required to inhibit 50% of trypsin activity, under the trypsin inhibition assay.

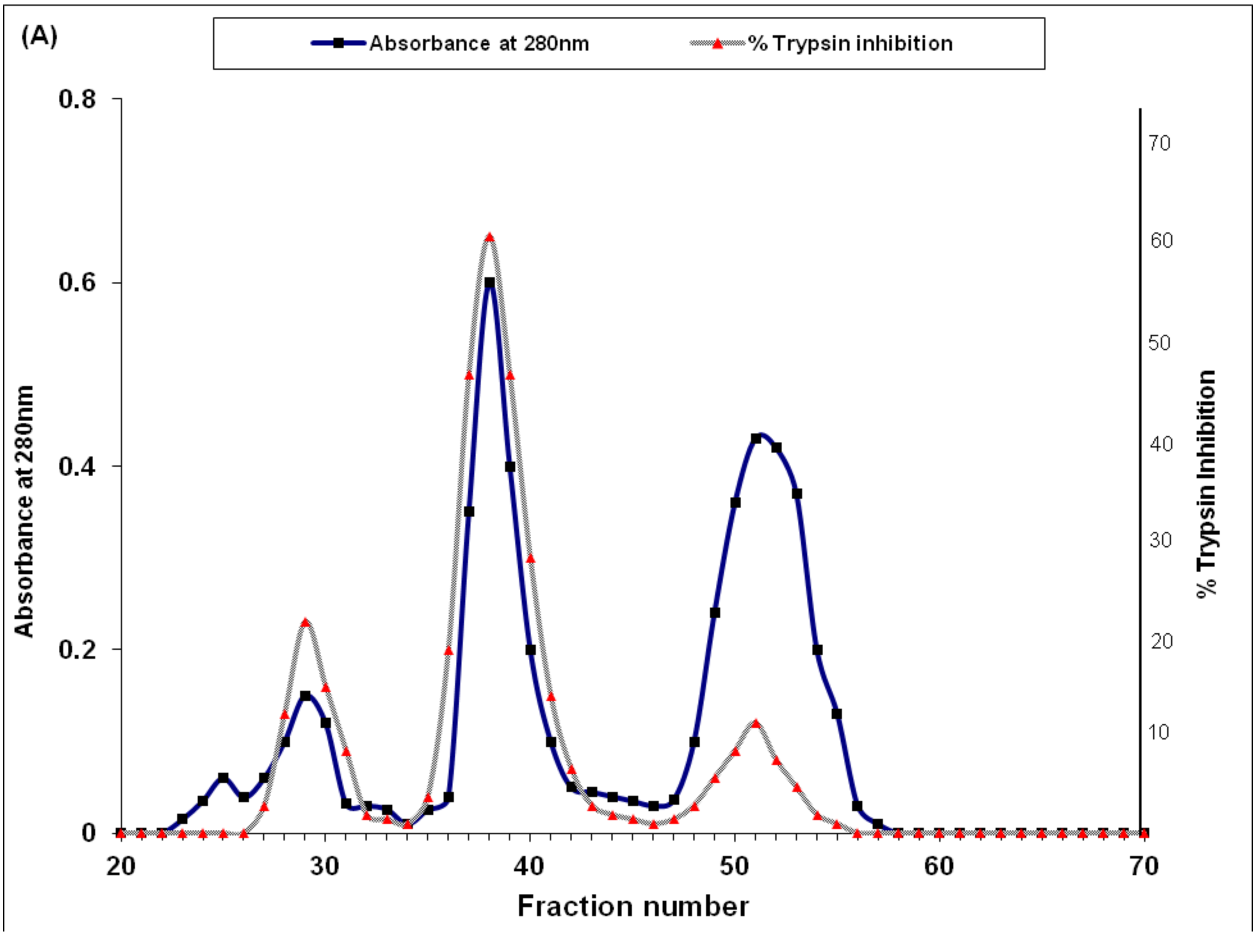
(A)
Absorbance at 280nm
% Trypsin inhibition
0.8
0.6
0.4
0.2
0
Absorbance at 280nm
70
60
50
40
30
20
10
% Trypsin Inhibition
20
30
40
50
60
70
Fraction number

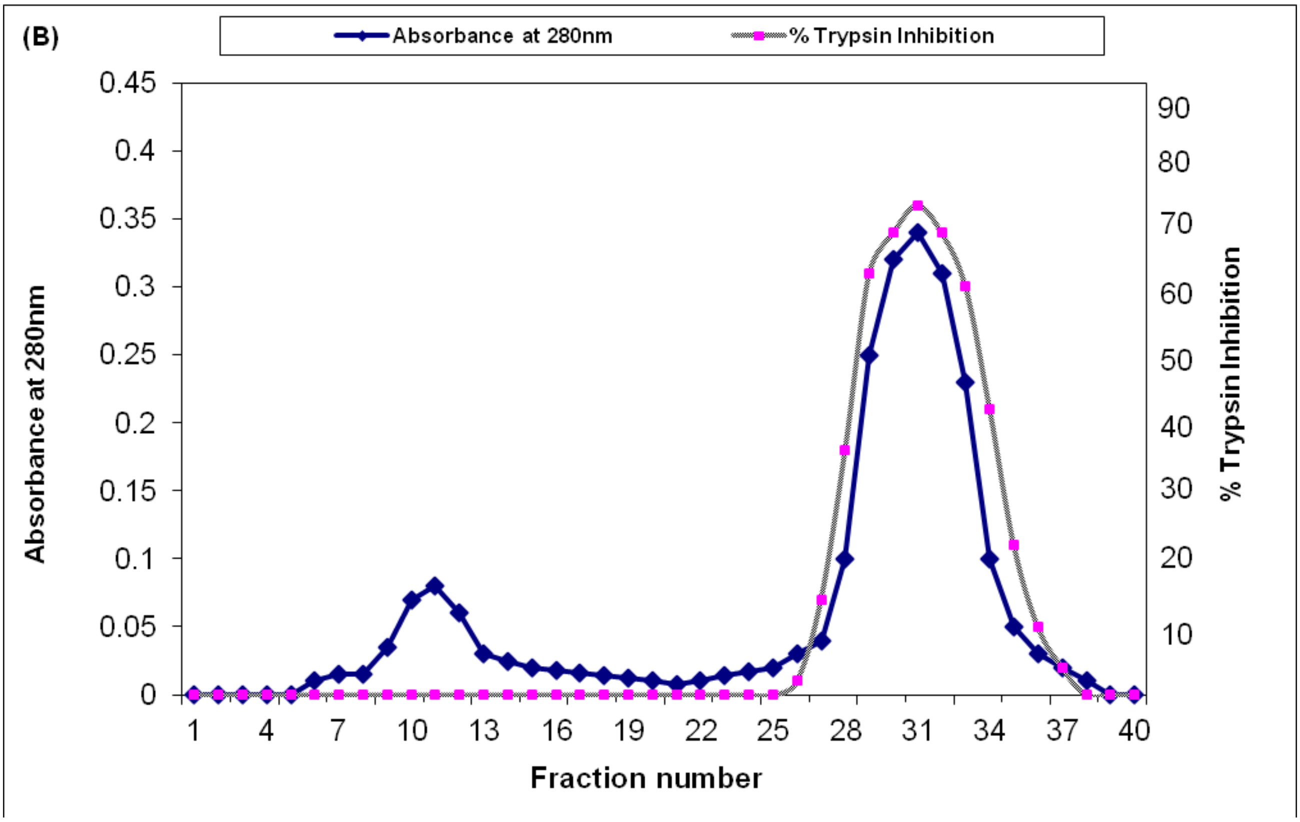

Figure 1 (A) Sephadex G-75 gel filtration chromatography of ammonium sulfate precipitated fraction (F_2) of trypsin inhibitor from the seeds of *Madhuca indica*. **(B)** Fractions with trypsin-inhibiting activity, purified from gel filtration column were subjected to trypsin-sepharose affinity column.

Table 2: Development of *H. armigera* at different concentration fed on diet incorporating MiTI (% w/w).

Dosage (% w/w)	Pupation %	% adult emergence	Mean Deformities (%)	Mean Fecundity (%) (n=10)
Without Inhibitor (Control)	95.4±0.13	93.5±0.16	05.3±0.14	98.3±0.25
0.5% MiTI	83.4±0.15	76.4±0.19	25.8±0.17	81.6±0.14
1.0% MiTI	78.3 ±0.14	69.8±0.13	37.1±0.13	62.5±0.13
1.5% MiTI	67.2±0.14	61.2±0.15	52.3±0.16	49.4±0.24

Table 3 Nutritional indices of Helicoverpa armigera larvae reared on 1 %w/w MiTI supplemented diet.

**Parameter (Mean ± SE)**									
**Instar stage**	**Dosage**	**I (mg)**	**F (mg)**	**ΔB (mg)**	**CI%**	**AD%**	**ECI%**	**ECD%**	**MC**
**Fourth instar**	**Control**	94.43±5.19	41.66±4.13	21.56±1.89	47.69±1.17	55.88±1.25	22.83±0.78	40.86±1.34	59.14±1.85
	**1 %w/w MiTI**	83.69±6.07	32.42±3.34	8.04±1.18	67.87±1.54	61.26±1.97	9.60±0.84	15.68±0.97	84.32±1.26
**Fifth instar**	**Control**	106.60±8.38	64.62±7.01	14.66±1.07	32.59±0.93	39.38±0.84	13.75±0.29	34.92±0.98	65.08±1.27
	**1 %w/w MiTI**	91.62±7.57	53.00±4.17	7.14±0.14	58.36±1.04	42.52±0.57	7.79±0.14	18.49±0.48	81.51±1.91

I= weight of food consumed, F= weight of feces produced during the feeding period, ΔB= change in body weight, CI= consumption index, AD= approximate digestibility, ECI= efficiency of conversion of ingested food

ECD= efficiency of conversion of digested food, CM= Metabolic cost

ECI= (ΔB/I) ×100; ECD= [ΔB/ (I−F)] ×100; CI= (I/A) ×100 and AD [(I−F)/I] ×100. The metabolic cost (MC) was calculated as 100−ECD.
